# Schizophrenia and Mega cisterna magna: a Case report

**DOI:** 10.1192/j.eurpsy.2023.2230

**Published:** 2023-07-19

**Authors:** E. E. Güneysu, B. Başyiğit Sayğılı

**Affiliations:** Psychiatry, Erenkoy Mental And Nervous Diseases Training And Research Hospital, Istanbul, Turkey, Istanbul, Türkiye

## Abstract

**Introduction:**

Mega cisterna magna is a developmental malformation of the posterior fossa, the cisterna magna is larger than normal, and the vermis and cerebellar hemispheres are morphologically normal. (Zimmer EZ et al. Obstet 2007; 276:487-490.) Although the relationship between this anomaly and psychiatric disorders is emphasized, its nature has not fully understood.

**Objectives:**

In this abstract, we report a case of schizophrenia with mega cisterna magna. We aimed to draw attention to the relationship between congenital malformations and schizophrenia since studies on congenital malformations were mostly conducted with epilepsy in the literature. (Lishman’s Organic Psychiatry: A Textbook of Neuropsychiatry, Fourth Edition, Chapter 6, 2009.)

**Methods:**

The patient is a 28-year-old male, single, secondary school graduate and unemployed. The patient known to has used volatile substance, cannabinoids and synthetic cannabinoids between the ages of 15-22 and has a psychiatric history of approximately 8 years. He had a total of 5 hospitalizations, the last of which was in our clinic 2 years ago. The patient, who was known to have no substance use for 6 years, had negative symptoms for about 4 years. According to the information received from the patient’s relatives, he was admitted to our clinic with complaints of decreased mobility, decreased communication, refusal to eat and drink, decreased sleep, self-talk, standing for a long time and looking at a single point; which had started in the last 10 days after non-compliance of treatment for the last 3 weeks.

**Results:**

The physical and the neurologic examinations were unremarkable. In the psychiatric examination, he was conscious, oriented, and cooperative. Self-care and psychomotor activity were decreased. His mood and affect were dysphoric and limited. His speech rate, spontaneity and intonation were decreased. His thought content couldn’t be evaluated properly because of the mutism. His attention was decreased. Laboratory studies were unremarkable. Non-contrast brain CT and MRI showed an appearance compatible with mega cisterna magna in the mid-left parasagittal area in the retrocerebellar region. There was a history of staying in NICU for 8 days when he was a newborn. There was no family history of psychiatric illness.

**Conclusions:**

The relationship between psychosis and clinical significance of MCM has not defined completely yet. Although the case we selected is rarely seen, there is one more example in the literature.(Karayilan S et al. Anatolian Psychiatry Journal; Sivas Vol. 14, Iss. 1, (Mar 2013):90-92.) Maybe the reason why there is so limited information in the literature on this topic is that congenital malformations are presented at a much younger age than psychosis with neurological diseases such as epilepsy. In conclusion, perhaps more detailed clinical follow-ups of these cases will enable new reports.

**Disclosure of Interest:**

None Declared

